# Coinfections in the lung: How viral infection creates a favorable environment for bacterial and fungal infections

**DOI:** 10.1371/journal.ppat.1011334

**Published:** 2023-05-04

**Authors:** Joshua J. Obar, Kelly M. Shepardson

**Affiliations:** 1 Geisel School of Medicine at Dartmouth, Department of Microbiology & Immunology, Lebanon, New Hampshire, United States of America; 2 University of California, Merced, Department of Molecular and Cell Biology, Merced, California, United States of America; University of Maryland, Baltimore, UNITED STATES

## Introduction

While it is well established that secondary bacterial pneumonia plays a significant role in the morbidity and mortality of patients with severe respiratory viral infections, bacteria are not the only threat for these patients. Over the last decade, there has been a rise in the number of patients with severe respiratory viral infections acquiring secondary fungal infections, specifically with the filamentous mold *Aspergillus fumigatus* [1,2]. Importantly, while secondary fungal infections occur less frequently than secondary bacterial pneumonias, they are associated with greater mortality [1]. Because immune suppression is the major risk factor for acquiring fungal infections [3], severe respiratory viral infections may create a transient immune suppressed state allowing for these secondary infections to occur. Here, we discuss what we have learned about how antiviral host responses create a lung environment susceptible to bacterial infection and how this may translate into fungal susceptibility.

## Viral infection “aftershocks”

**Fig 1:**
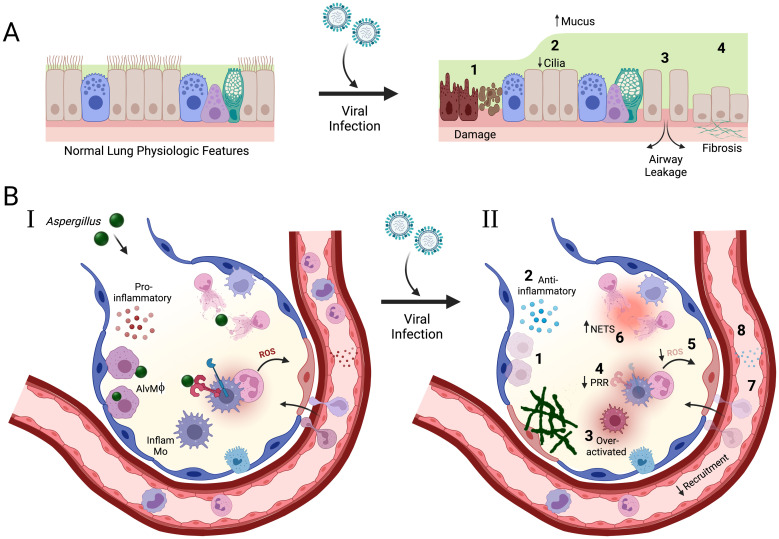
Graphic of the major physiologic and immunologic consequences from viral infection found to be involved in secondary bacterial susceptibility described in [4]. (**A**) The physiological consequences from viral infection include (1) epithelial damage/death (morphology changes, apoptosis), (2) airway function (loss of cilia, increased mucus), (3) airway leakage (loss of tight junctions), and (4) delayed repair (fibrosis). (**B**) Normal antifungal immune response to *Aspergillus* conidia (I) may be altered by respiratory viral infection leading to invasive disease (fungal biofilm formation) (II). The immunological consequences from viral infection include (1) depletion of alveolar macrophages, (2) anti-inflammatory skewed, (3) over-activated inflammatory monocytes, (4) recognition (decreased PRRs), (5 and 6) antifungal effector mechanisms (decreased ROS, increased NETs), (7 and 8) decreased cell recruitment and chemokines/cytokines. This figure was exported under a paid subscription. Created with BioRender.com.

Many of the physiologic and immunologic outcomes in the lung resulting from respiratory viral infection, especially with influenza viruses, are well established as to their role in secondary bacterial infection susceptibility and beginning to be elucidated for secondary fungal infections. Without going into great depth, as these were just recently reviewed elsewhere [4], Fig 1 portrays some of these physiological and immunological mechanisms altered in the lungs. The physiological consequences from respiratory viral infection include epithelial damage (morphology changes, leakage, apoptosis), altered airway function (loss of cilia, increased mucus), and delayed repair (fibrosis) (Fig 1A). The immunological consequences from respiratory viral infections are slightly more complicated, but include recognition (decreased pattern recognition receptors), inflammation (anti-inflammatory skewed), cell function (decreased phagocytosis, impaired ROS production, inhibition of activation), and changes in cell type (loss of alveolar macrophages, hyperactivated inflammatory monocytes, decreased leukocyte recruitment) (Fig 1B). Recently, a transcriptional analysis of human lower respiratory tract samples from influenza-associated pulmonary aspergillosis (IAPA) patients suggested there is a three-level breach in antifungal immunity, including phagocytosis, killing of conidia/hyphae, and epithelial damage [5]. While this study provides a beginning foundation of the antiviral outcomes clinically associated with IAPA, there remains many unknowns. Thus, we will describe different aspects from these outcomes and how they may be involved in creating a lung environment permissive to fungal infection.

### Aspect 1: Physiological

A key fundamental difference between the bacteria associated with secondary infections (*Staphylococcus* spp. and *Streptococcus* spp.) [6] and *Aspergillus* spp. is that molds are saprophytic organisms. Survival and growth of molds rely upon degradation of their surrounding environment, indicating their ability to grow in harsh conditions. Proteases and other secondary metabolites important for nutrient acquisition by *Aspergillus* spp. have been shown to be necessary for pathogenesis [7,8]. Many of the antiviral responses described above (Fig 1B) contribute to creating damage, mostly physical in nature, within the lung. This damaged, “decomposed-like” environment likely contributes to the success of *Aspergillus* spp. in the post-viral infection lung, supported by the observation of fungal growth occurring near epithelial damage in patients with COVID-19-associated pulmonary aspergillosis (CAPA) [5] and IAPA [9]. Specifically, nutrients are easier to obtain due to both the death of epithelial and immune cells together with vasculature and epithelial leakage, making macromolecules (sugars, amino acids) and essential nutrients (iron and zinc [10]) more available. Beyond the access to “free” nutrients, the ability to adapt to changing conditions plays a major role in *Aspergillus* spp. being able to establish an infection. Damage and inflammation in the lung leads to decreased oxygen availability [4]. *Aspergillus* spp. in general, but specifically known for *A*. *fumigatus*, adapt quickly and efficiently to low oxygen environments [11,12]. This adaptation helps thwart immune attacks and resist antifungal drugs due to cell wall architecture changes that make it harder to penetrate and harm *A*. *fumigatus* [11]. Additionally, *A*. *fumigatus* can grow under low oxygen conditions [12], providing another edge in this environment. Overall, from what is known about the damaged lung environment resulting from a viral infection, this suggests that it provides a prime environment for the establishment and growth of fungi.

### Aspect 2: Immunological

It is not surprising that many of the antibacterial innate mechanisms rendered nonfunctional or skewed because of a primary viral infection (Fig 1) can also be involved in secondary fungal infection susceptibility, as innate mechanisms are generally nonspecific. However, there are many aspects of fungi that are not shared with the bacteria associated with secondary infections. Some unique differences include the transition of fungal organisms in the host into different morphological states and, importantly, that fungi have unique surface components used by the host for recognition [13–15]. These aspects influence the host immunological response and so here, we will discuss how these differences may have an impact on secondary fungal infection susceptibility as it relates to *Aspergillus*.

We can think of infections with *Aspergillus* spp. as a sprint between the innate immune system and the growth of the fungus. *Aspergillus* spp. transition through 3 major growth stages during infection, starting at resting, dormant conidia, transitioning to swollen conidia, and finally germinating into hyphae forming a fungal biofilm. There are tools the immune system uses for each growth stage, but ultimately, preventing invasive hyphal growth is the most important for survival. This is most effectively accomplished by killing the conidia. Alveolar macrophages act as the first line of defense in the lung airways, ingesting and killing *Aspergillus* conidia. When alveolar macrophages are functionally impaired due to corticosteroid treatment [16], loss of NADPH oxidase activity [8], or loss of MAVS signaling [17] it results in increased susceptibility to *A*. *fumigatus* challenge. Respiratory virus infection can mediate depletion of alveolar macrophages [4] that may provide an opportunity for *Aspergillus* spp. to get a foothold in the lung.

Recruited inflammatory monocytes and neutrophils are critical for control and clearance of conidia during *A*. *fumigatus* infection [18]. Based on what we know from secondary bacterial infections, decreased alveolar macrophages, disrupted epithelium, and dampened inflammation affects activation and recruitment of these effector cells [4]. This is likely true for secondary fungal infections as lung epithelial cells play an important role in protection to *Aspergillus* infections through induction of chemokines that lead to efficient recruitment of these cells [19]. Pro-inflammatory mediators, such as IL-1α, IL-1β, TNFα, and CXCL1, specifically produced by the lung epithelium, are required for providing protection from *Aspergillus* spp. infections, via cell activation and recruitment, and in their absence, invasive disease and mortality occur [20]. Therefore, damaged/dead epithelium as a result of viral infections is also likely to contribute to immunological deficiencies (not just physiological) leading to fungal susceptibility. Recently, it was found that decreased neutrophil recruitment following *A*. *fumigatus* infection in mice post-influenza resulted in increased susceptibility [21], but others have not observed this in mice [22] or humans [5,23], suggesting further defects in antifungal immunity must exist following viral infection.

Concerning antifungal mediators, our recent data demonstrates that antifungal killing is decreased following influenza infection [22]. Mechanistically, both ROS-dependent and -independent pathways have been shown to be critical for host resistance against *A*. *fumigatus*, both of which can be impaired by viral infection [4,20]. ROS production and phagocytosis from inflammatory monocytes [18] and neutrophils is important for preventing conidial germination into hyphae, upon which phagocytosis becomes impossible. Neutrophil NETosis can also act as an antifungal mechanism for controlling *Aspergillus* hyphae [24]. However, increased production of NETs from post-viral neutrophils may add to the damage, as found with acute lung injury models [25], rather than enhancing clearance of *Aspergillus* during secondary infection. Importantly, we demonstrated that ROS-independent mechanisms of antifungal immunity are impacted by prior viral infection [22], specifically impaired phagolysosome maturation. Supporting these murine model results, Feys and colleagues found impaired neutrophil degranulation and LC3-phagolysosome-associated transcriptional signature in humans with IAPA [5]. Additionally, B1a-dependent natural IgG antibodies were found to mediate protection via neutrophil phagocytosis of *Aspergillus*, during both IAPA and CAPA, but also following corticosteroid treatment [23].

Decreased levels of PRRs following a viral infection, including multiple TLRs and RIG-I, are associated with poor outcome to secondary bacterial infections [4]. We, and others, have demonstrated that there is transcriptional down-regulation of TLR9/Irak4 [22] and TLR2 [5] in mice and humans with IAPA, respectively. Although these PRRs have a role in antifungal immunity [20], it remains unknown whether there is a direct role during IAPA. Additionally, C-type lectin receptors (CLRs) are critical for protection and survival from fungal infections [26]. CLRs distinguish conserved PAMPs found in the cell wall of fungal pathogens, eliciting antifungal-specific innate immune responses, and shaping the adaptive immune response. An ex vivo infection model of human alveolar macrophages found decreased expression of CLEC7A, an essential CLR required for a protective antifungal response, following influenza infection [27]. However, there remains very little known about what happens to CLR signaling during influenza infection and its involvement in IAPA.

## Perspectives

Importantly, many of the classical clinical risk factors normally associated with an elevated risk of patients developing Aspergillosis [3] are not common among the patients acquiring IAPA/CAPA. This results in decreased testing, misdiagnosis, and delayed antifungal treatments. For the patient, this could be detrimental because delayed treatment may provide *Aspergillus* spp. time to grow into a more robust biofilm, which is more resistant to the limited antifungal drugs available for treatment. While we do not know the full scope of occurrence of IAPA/CAPA, understanding how these *Aspergillus* spp. infections occur is critically important. As described here, many of the aspects from viral infections have implications in antifungal immunity. It is likely that susceptibility to IAPA requires a combination of these physiologic and immunologic antiviral outcomes. Further, with high mortality rates (40% to 90%) and limited, and toxic antifungal treatments [3], it is imperative that we find ways to prevent and treat IAPA [9].

Because this is a relatively new and expanding research field, there remains much to be discovered about why viral infections lead to fungal susceptibility. One important area of research that needs to be addressed is determining how data from clinical cohort studies, including the gene expression data described above, translates into post-viral fungal pneumonia susceptibility. Here, the recently developed mouse models of IAPA [21–23] will be critical for our basic mechanistic understanding of IAPA, as well as providing a preclinical model for interventional studies. Are there common antifungal mechanisms that are suppressed by viral infection (e.g., CLRs, TH17 responses, phagosome)? How does the environment of a post-viral lung contribute (e.g., epithelial damage, nutrients) to susceptibility to fungal challenge? Because both Influenza and SARS-CoV-2 act as a risk factor, are there viral-specific immune suppression mechanisms? Additionally, due to the heterogeneity of *A*. *fumigatus* strains, an important question that remains open is, are susceptibility and outcome strain dependent [28]? Finally, how can antiviral, antifungal, and anti-inflammatory treatments impact the IAPA outcomes needs to be more thoroughly explored, as early amphotericin B treatment [29] or oseltamivir [9] treatment improved clinical outcomes in preclinical models. Overall, we need to expand our knowledge of the host–fungal interaction in this post-viral environment to foster the development of fungal- and host-directed therapies.
